# Literary Notes

**Published:** 1909-01-02

**Authors:** 


### Literary Notes.

Mr. Edward Arnold has just published a new revised and enlarged edition of "The Diagnosis of Nervous Diseases," by Dr. Purves Stewart. The text has been revised throughout, and several chapters with a number of fresh illustrations have been added. The first edition was published two years ago.

A work entitled the "Treasury of Human Inheritance" is shortly to be issued by the Francis Galton Laboratory for National Eugenics. It will be published under the auspices of the University of London by Messrs. Dulau, and the subscription to each set of four parts will be 20s., or for each part alone 7s. This proposed publication by the Eugenics Laboratory cannot fail to be of the highest value to those interested in general science and in scientific medicine. To have at hand carefully collected pedigrees, in which the recurrence of disease or any other abnormality is shown, will be useful not merely for reference but as a basis for important future deductions. It is also gratifying to note that all controversial matter, involving a discussion of theories, will be avoided, and that the genealogical trees will be explained simply and solely in so far as concerns the facts. We indeed welcome such a decisive step towards the scientific explanation of the vexed question of inheritance.

